# Near-optimal assembly for shotgun sequencing with noisy reads

**DOI:** 10.1186/1471-2105-15-S9-S4

**Published:** 2014-09-10

**Authors:** Ka-Kit Lam, Asif Khalak, David Tse

**Affiliations:** 1 Department of Electrical Engineering and Computer Sciences, UC Berkeley, Berkeley, California, United States; 2 Pacific Biosciences, Menlo Park, California, United States Full list of author information is available at the end of the article

**Keywords:** De novo sequence assembly, genome finishing, methods for emerging sequencing technologies

## Abstract

Recent work identified the fundamental limits on the information requirements in terms of read length and coverage depth required for successful *de novo *genome reconstruction from shotgun sequencing data, based on the idealistic assumption of no errors in the reads (noiseless reads). In this work, we show that even when there is noise in the reads, one can successfully reconstruct with information requirements close to the noiseless fundamental limit. A new assembly algorithm, X-phased Multibridging, is designed based on a probabilistic model of the genome. It is shown through analysis to perform well on the model, and through simulations to perform well on real genomes.

## Background

Optimality in the acquisition and processing of DNA sequence data represents a serious technology challenge from various perspectives including sample preparation, instrumentation and algorithm development. Despite scientific achievements such as the sequencing of the human genome and ambitious plans for the future [1,2], there is no single, overarching framework to identify the fundamental limits in terms of information requirements required for successful output of the genome from the sequence data.

Information theory has been successful in providing the foundation for such a framework in digital communication [3], and we believe that it can also provide insights into understanding the essential aspects of DNA sequencing. A first step in this direction has been taken in the recent work [4], where the fundamental limits on the minimum read length and coverage depth required for successful assembly are identified in terms of the statistics of various repeat patterns in the genome. Successful assembly is defined as the reconstruction of the underlying genome, i.e. genome finishing [5]. The genome finishing problem is particularly attractive for analysis because it is clearly and unambiguously defined and is arguably the ultimate goal in assembly. There is also a scientific need for finished genomes [6,7]. Until recently, automated genome finishing was beyond reach [8] in all but the simplest of genomes. New advances using ultra-long read single-molecule sequencing, however, have reported successful automated finishing [9,10]. Even in the case where finished assembly is not possible, the results in [4] provide insights on optimal use of read information since the heart of the problem lies in how one can optimally use the read information to resolve repeats.

**Figure 1:**
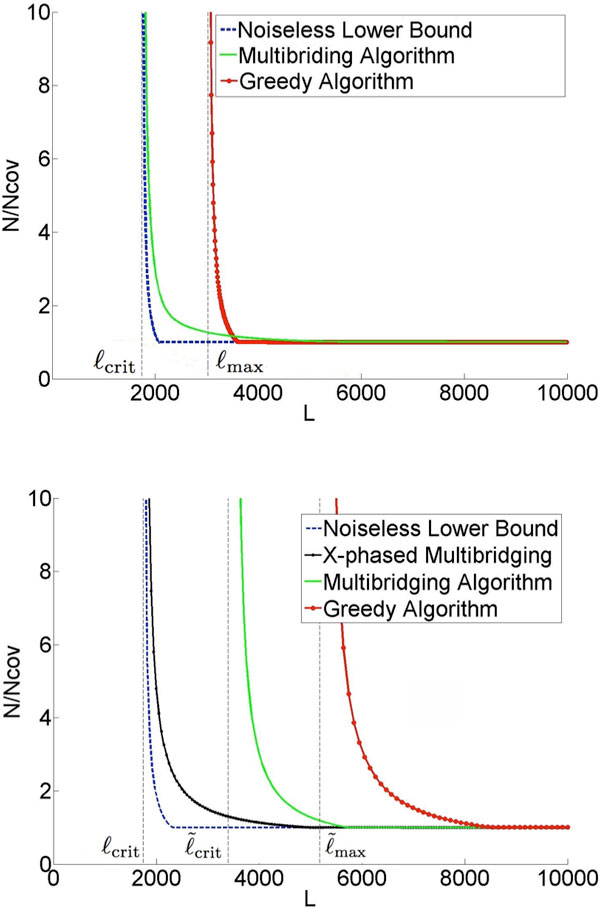
**Information requirement to reconstruct *E. coli *K12**. ℓ_crit _= 1744, ${\tilde{\ell }}_{\text{crit}}=3393$

Figure 1a gives an example result for the repeat statistics of *E. coli *K12. The x-axis of the

plot is the read length and the y-axis is the coverage depth normalized by the Lander-Waterman depth (number of reads needed to cover the genome [11]). The lower bound identifies the necessary read length and coverage depth required for *any *assembly algorithm to be successful with these repeat statistics. An assembly algorithm called Multibridging Algorithm was presented, whose read length and coverage depth requirements are very close to the lower bound, thus tightly characterizing the fundamental information requirements. The result shows a critical phenomenon at a certain read length *L *=ℓ_crit_: below this critical read length, reconstruction is impossible no matter how high the coverage depth; slightly above this read length, reconstruction is possible with Lander-Waterman coverage depth. This critical read length is given byℓ_crit _= max{ℓ_int_,ℓ_tri_}^, ^whereℓ_int _is the length of the longest pair of exact interleaved repeats andℓ_tri _is the length of the longest exact triple repeat in the genome, and has its roots in earlier work by Ukkonen on Sequencing-by-Hybridization [12]. The framework also allows the analysis of specific algorithms and the comparison with the fundamental limit; the plot shows for example the performance of the Greedy Algorithm and we see that its information requirement is far from the fundamental limit.

A key simplifying assumption in [4] is that there are no errors in the reads (noiseless reads). However reads are noisy in all present-day sequencing technologies, ranging from primarily substitution errors in Illumina ^® ^platforms, to primarily insertion-deletion errors in Ion Torren ^® ^and PacBio ^® ^platforms. The following question is the focus of the current paper: in the presence of read noise, can we still successfully assemble with a read length and coverage depth close to the minimum in the noiseless case? A recent work [13] with an existing assembler suggests that the information requirement for genome finishing substantially exceeds the noiseless limit. However, it is not obvious whether the limitations lie in the fundamental effect of read noise or in the sub-optimality of the algorithms in the assembly pipeline.

## Results

The difficulty of the assembly problem depends crucially on the genome repeat statistics. Our approach to answering the question of the fundamental effect of read noise is based on design and analysis using a parametric probabilistic model of the genome that matches the key features of the repeat statistics we observe in genomes. In particular, it models the presence of long flanked repeats which are repeats flanked by statistically uncorrelated region. Figure 1b shows a plot of the predicted information requirement for reliable reconstruction by various algorithms under a substitution error rate of 1%. The plot is based on analytical formulas derived under our genome model with parameters set to match the statistics of *E. coli *K12. We show that it is possible in many cases to develop algorithms that approach the noiseless lower bound even when the reads are noisy. Specifically, the X-phased Multibridging Algorithm has close to the same critical read length *L *=ℓ_crit _as in the noiseless case and only slightly greater coverage depth requirement for read lengths greater than the critical read length.

**Figure 2:**
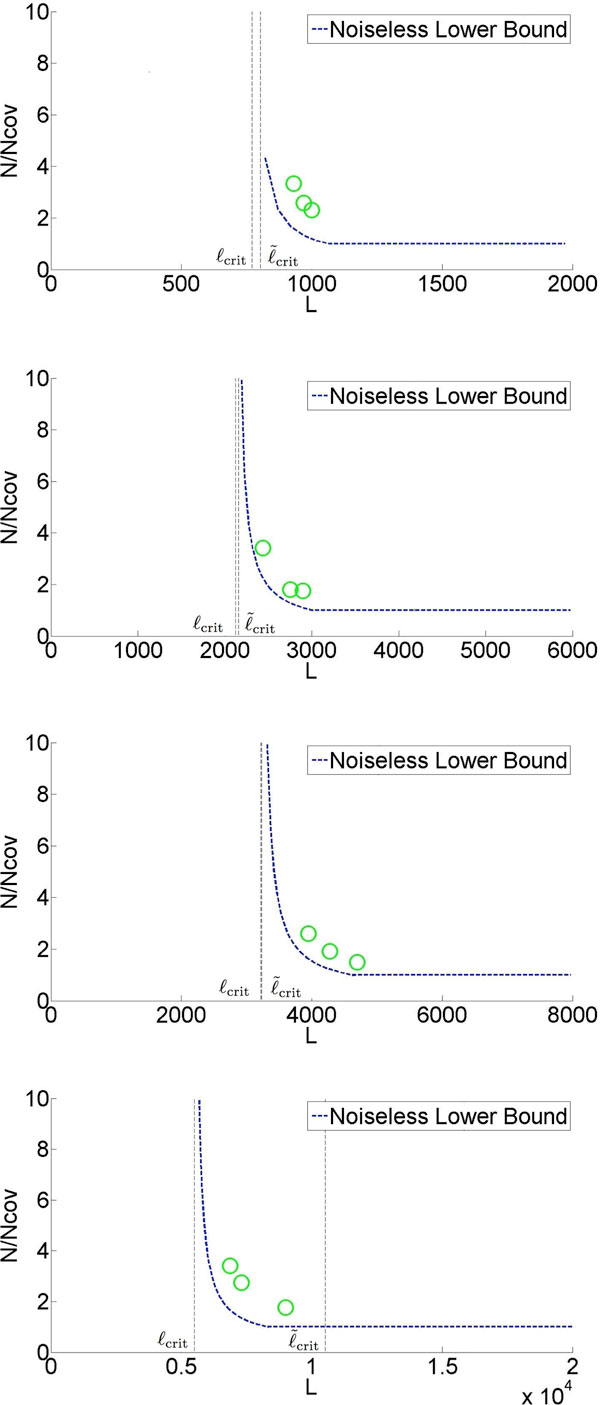
**Simulation results on a prototype assembler (substitution noise of rate 1**.5 %)

We then proceed to build a prototype assembler based on the analytical insights and we perform experiments on real genomes. As shown in Figure 2, we test the prototype assembler by using it to assemble noisy reads sampled from 4 different genomes. At coverage and read length indicated by a green circle, we successfully assemble noisy reads into one contig (in most cases with more than 99% of the content matched when compared with the ground truth). Note that the information requirement is close to the noiseless lower bound. Moreover, the algorithm (X-phased Multibridging) is computationally effisscient with the most computational expensive step being the computation of overlap of reads/K-mers, which is an unavoidable procedure in most assembly algorithms.

The main conclusion of this work is that, with an appropriately designed assembly algorithm, the information requirement for genome assembly is surprisingly insensitive to read noise. The basic reason is that the redundancy required by the Lander-Waterman coverage constraint can be used to denoise the data. This is consistent with the asymptotic result obtained in [14] and the practical approach taken in [9]. However, the result in [14] is based on a very simplistic i.i.d. random genome model, while the model and genomes considered in the present paper both have long repeats. A natural extension of the Multibridging Algorithm in [4] to handle noisy reads allows the resolution of these long flanked repeats if the reads are long enough to span them, thus allowing reconstruction provided that the read length is greater than $L={\tilde{\ell }}_{\text{crit}}=\text{max}\left\{{\tilde{\ell }}_{\text{int}},{\tilde{\ell }}_{\text{tri}}\right\}$, where ${\tilde{\ell }}_{\text{int}}$ is the length of the longest pair of flanked interleaved repeats andℓ_tri _is the length of the longest flanked triple repeat in the genome. This condition is shown as a vertical asymptote of the "Multibridging Algorithm" curve in Figure 1b. By exploiting the redundancy in the read coverage to resolve read errors, the X-phased Multibridging can phase the polymorphism across the flanked repeat copies using only reads that span the exact repeats. Hence, reconstruction is achievable with a read length

close to *L *=ℓ_crit_,s which is the noiseless limit.

### Related work

All assemblers must somehow address the problem of resolving noise in the reads during genome reconstruction. However, the traditional approaches to measuring assembly performance makes quantitative comparisons challenging for unfinished genomes [15]. In most cases, the heart of the assembly problem lies in processing of the assembly graph, as in [16-18]. A common strategy for dealing with ambiguity from the reads lies in filtering the massively parallel sequencing data using the graph structure prior to traversing possible assembly solutions. In the present work, however, we are focused on the often-overlooked goal of optimal data efficiency. Thus, to the extent possible we distinguish between the read error and the mapping ambiguity associated with the shotgun sampling process. The proposed assembler, X-phased Multibridging, adds information to the assembly graph based on a novel analysis of the underlying reads.

## Methods

The path towards developing X-phased Multibridging is outlined as follows.

1 Setting up the shotgun sequencing model and problem formulation.

2 Analyzing repeats structure of genome and their relationship to the information requirement for genome finishing.

3 Developing a parametric probabilistic model that captures the long tail of the repeat statistics.

4 Deriving and analyzing an algorithm that require minimal information requirements for assembly -close to the noiseless lower bound.

5 Performing simulation-based experiments on real and synthetic genomes to characterize the performance of a prototype assembler for genome finishing.

6 Extending the algorithm to address the problem of indel noise.

## Shotgun sequencing model and problem formulation

### Sequencing model

Let **s **be a length *G *target genome being sequenced with each base in the alphabet set Σ = {*A, C, G, T*}. In the shotgun sequencing process, the sequencing instrument samples *N *reads, ${\vec{r}}_{1}$,..., ${\vec{r}}_{N}$ of length *L *and sampled uniformly and independently from **s**. This unbiased sampling assumption is made for simplicity and is also supported by the characteristics of single-molecule (e.g. PacBio ^®^) data. Each read is a noisy version of the corresponding length *L *substring on the genome. The noise may consist of base insertions, substitutions or deletions. Our analysis focus on substitution noise first. In a later section, indel noise is addressed. In the substitution noise model, let *p *be the probability that a base is substituted by another base, with probability *p/*3 to be any other base. The errors are assumed to be independent across bases and across reads.

### Formulation

Successful reconstruction by an algorithm is defined by the requirement that, with probability at least 1 *- ϵ*, the reconstruction $\hat{\text{s}}$ is a single contig which is within edit distance *δ *from the target genome **s**. If an algorithm can achieve that guarantee at some (*N, L*), it is called *ϵ*-feasible at (*N, L*). This formulation implies automated genome finishing, because the output of the algorithm is one single contig. The fundamental limit for the assembly problem is the set of (*N, L*) for which successful reconstruction is possible by some algorithms. If $\hat{\text{s}}$ is directly spelled out from a correct placement of the reads, the edit distance between  ŝ and **s **is of the order of *pG*, where the error rate is *p*. This motivates fixing *δ *= 2*pG *for concreteness. The quality of the assembly can be further improved if we follow the assembly algorithm with a consensus stage in which we correct each base, e.g. with majority voting. But the consensus stage is not the focus in this paper.

## Repeats structure and their relationship to the information requirement for successful reconstruction

### Long exact repeats and their relationship to assembly with noiseless reads

We take a moment to carefully define the various types of exact repeats. Let ${\text{s}}_{\text{t}}^{\ell }$ denote the length-*ℓ *substring of the DNA sequence **s **starting at position *t*. An exact repeat of length *ℓ *is a substring appearing twice, at some positions *t*_1_, *t*_2 _(so ${\text{s}}_{{\text{t}}_{\text{1}}}^{\ell }\;\text{ = }\;{\text{s}}_{{\text{t}}_{\text{2}}}^{\ell }$) that is maximal (i.e. *s*(*t*_1 _*- *1) ≠ *s*(*t*_2 _*- *1) and *s*(*t*_1 _+ℓ) ≠ *s*(*t*_2 _+ℓ)).

Similarly, an exact triple repeat of length-*ℓ *is a substring appearing three times, at positions *t*_1_, *t*_2_, *t*_3_, such that ${\text{s}}_{{\text{t}}_{\text{1}}}^{\ell }\text{ = s}{\;}_{{\text{t}}_{\text{2}}}^{\ell }{\text{ = s}}_{{\text{t}}_{\text{3}}}^{\ell }$, and such that neither of *s*(*t*_1_*-*1) = *s*(*t*_2 _*- *1) = *s*(*t*_3_*-*1) nor *s*(*t*_1_+ℓ) = *s*(*t*_2_+ℓ) = *s*(*t*_3 _+ℓ) holds.

A copy of a repeat is a single one of the instances of the substring appearances. A pair of exact repeats refers to two exact repeats, each having two copies. A pair of exact repeats, one at positions *t*_1_, *t*_3 _with *t*_1 _<*t*_3 _and the second at positions *t*_2_, *t*_4 _with *t*_2 _<*t*_4_, is interleaved if *t*_1 _*< t*_2 _*< t*_3 _*< t*_4 _or *t*_2 _*< t*_1 _*< t*_4 _*< t*_3_. The length of a pair of exact interleaved repeats is de-fined to be the length of the shorter of the two exact repeats. A typical appearance of a pair of exact interleaved repeat is -X-Y-X-Y- where × and Y represent two different exact repeat copies and the dashes represent non-identical sequence content.

We letℓ_max _be the length of the longest exact repeat,ℓ_int _be the length of the longest pair of exact interleaved repeats andℓ_tri _be the length of the longest exact triple repeat.

As mentioned in the introduction, it was observed that the read length and coverage depth required for successful reconstruction using noiseless reads for many genomes is governed by long exact repeats. For some algorithms (e.g. Greedy Algorithm), the read length requirement is bottlenecked byℓ_max_^. ^The Multi-bridging Algorithm in [4] can successfully reconstruct the genome with a minimum amount of information. The corresponding minimum read length requirement is the critical exact repeat lengthℓ_crit _= max(ℓ_int_,ℓ_tri_).

### Flanked repeats

**Figure 3:**
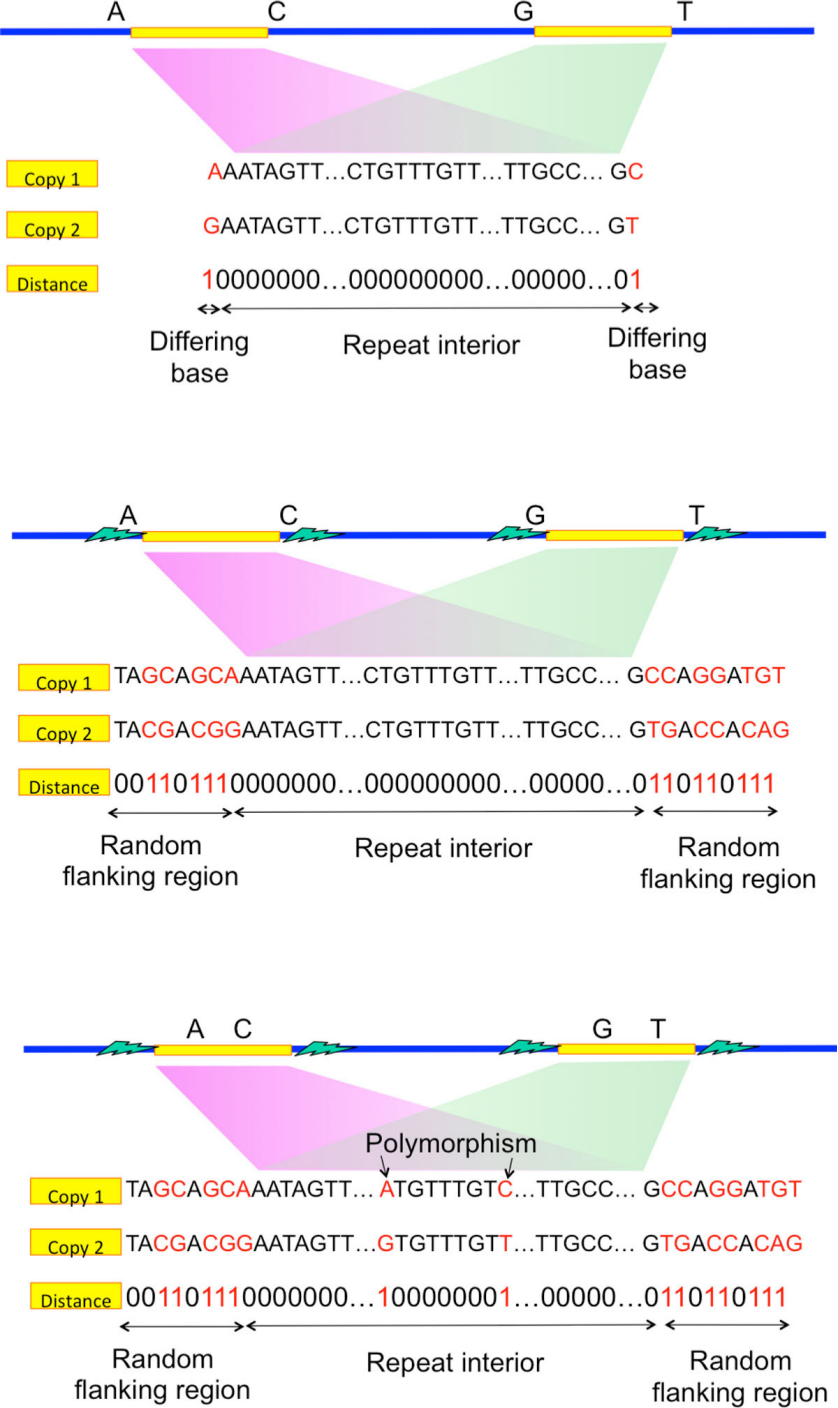
**Repeat pattern**.

While exact repeats are defined as the segments terminated on each end by a single differing base (Figure 3a), flanked repeats are defined by the segments terminated on each end by a statistically uncorrelated region. We call that ending region to be the *random flanking region*. A distinguishing characteristic of the random flanking region is a high Hamming distance to segment length ratio between the ends of two repeat copies. The ratio in the random flanking region is around 0.75, which matches with that when the genomic content is independently and uniformly randomly generated. We observe that long repeats of many genomes terminate with random flanking region. Additional statistical analysis is detailed in the Appendix.

If the repeat interior is exactly the same between two copies of the flanked repeat (Figure 3b), the corresponding flanked repeat is called a flanked exact repeat. If there are a few edits (called polymorphism) within the repeat interior (Figure 3c), the corresponding flanked repeat is called a flanked approximate repeat.

The length of the repeat interior bounded by the random flanking region is then the flanked repeat length. We let ${\tilde{\ell }}_{\text{max}}$ be the length of the longest flanked repeat, ${\tilde{\ell }}_{\text{int}}$ be the length of the longest pair of flanked interleaved repeats and ${\tilde{\ell }}_{\text{tri}}$ be the length of the longest flanked triple repeat. The critical flanked repeat length is then ${\tilde{\ell }}_{\text{crit}}=\text{max}\left({\tilde{\ell }}_{\text{int}\;},{\tilde{\ell }}_{\text{tri}}\right)$.

Long flanked exact repeats and their relationship to assembly with noisy reads

If all long flanked repeats are flanked exact repeats, we can utilize the information in the random flanking region to generalize Greedy Algorithm and Multi-bridging Algorithm to handle noisy reads. The corresponding information requirement is very similar to that when we are dealing with noiseless reads.

**Figure 4:**
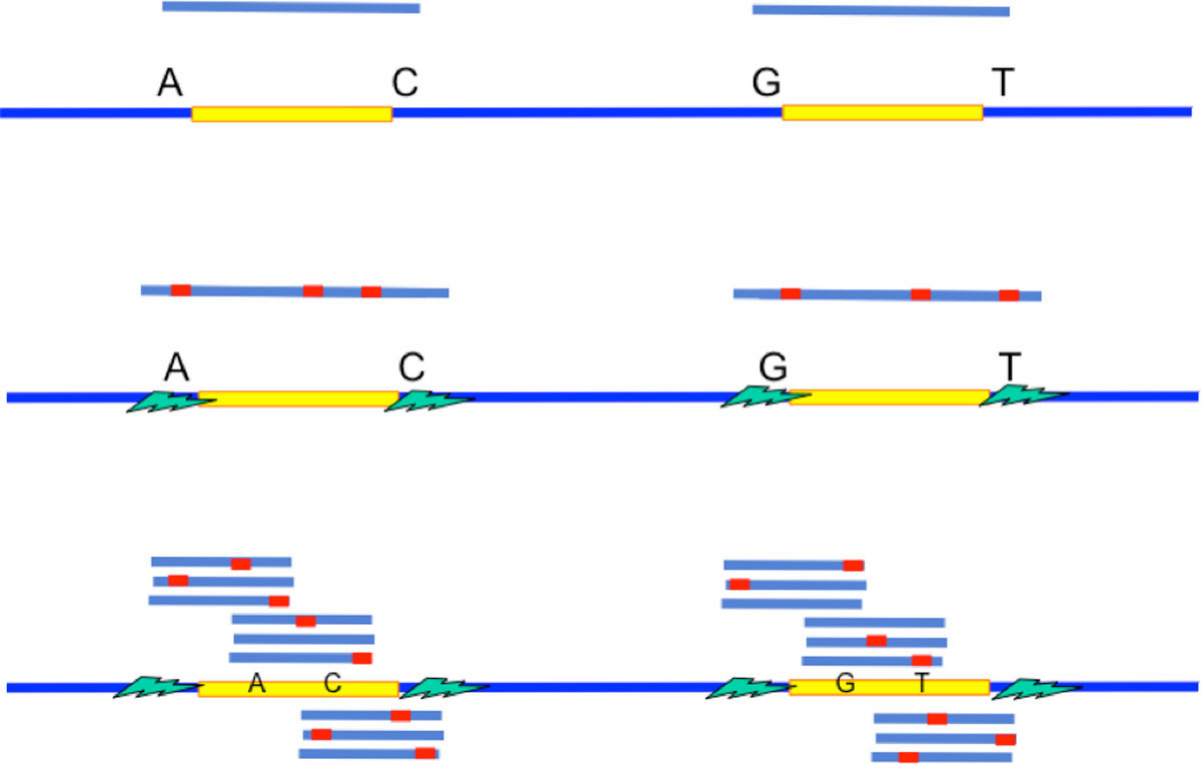
**Intuition behind the information requirement**.

The key intuition is as follows. A criterion for successful reconstruction is the existence of reads to span the repeats to their neighborhood. When a read is noiseless, it only need to be long enough to span the repeat interior to its neighborhood by one base (Figure 4a) so as to differentiate between two exact repeat copies. When a read is noisy, it then need to be long enough to span the repeat interior plus a short extension into the random flanking region (Figure 4b) so as to confidently differentiate between two flanked repeat copies. However, the Hamming distance between two flanked repeat copies' neighborhood in the random flanking region is very high even within a short length. This can be used to differentiate between two flanked repeat copies confidently even when the reads are noisy. The short extension into the random flanking region has a length which is typically of order of tens whereas the long repeat length is of order of thousands. Therefore, relative to the repeat length, the change of the critical read length requirement from handling noiseless reads to noisy reads is only marginal when all long repeats are flanked exact repeats.

Long flanked approximate repeats and their relationship to assembly with noisy reads

If a long flanked repeat is a flanked approximate repeat, the flanked repeat length may be significantly longer than the length of its longest enclosed exact repeat. Merely relying on the information provided by the random flanking region requires the reads to be of length longer than the flanked repeat length for successful reconstruction. This explains why the information requirement for Greedy Algorithm and Multibridging Algorithm has a significant increase when we use noisy reads instead of noiseless reads (as shown in Figure 1b). However, if we utilize the information provided by the coverage, we can still confidently differentiate different repeat copies by phasing the small edits within the repeat interior (Figure 4c). Specifically, we design X-phased Multibridging whose information requirement is close to the noiseless lower bound even when some long repeats are flanked approximate repeats, as shown in Figure 1b.

From information theoretic insight to algorithm design

Because of the structure of long flanked repeats, there are two important sources of information that we specifically want to utilize when designing data efficient algorithms to assemble noisy reads. They are

The random flanking region beyond the repeat interior

The coverage given by multiple reads overlapping at the same site

Greedy Algorithm(Alg 1) utilizes the random flanking region when considering overlap. The minimum read length needed for successful reconstruction is close to ${\tilde{\ell }}_{\text{max}}$.

Multibridging Algorithm(Alg 2) also utilizes the random flanking region but it improves upon Greedy Algorithm by using a De Bruijn graph to aid the resolution of flanked repeats. The minimum read length needed for successful reconstruction is close to ${\tilde{\ell }}_{\text{crit}}$.

X-phased Multibridging(Alg 3) further utilizes the coverage given by multiple reads to phase the polymorphism within the repeat interior of flanked approximate repeats. The minimum read length needed for successful reconstruction is close toℓ_crit_, which is the noiseless lower bound even when some long repeats are flanked approximate repeats.

## Model for genome

**Figure 5:**
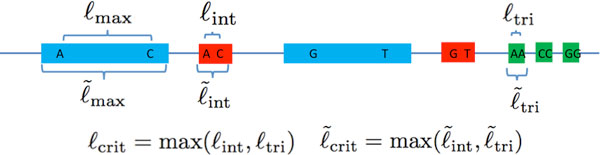
**Model for genome**.

To capture the key characteristics of repeats and to guide the design of assembly algorithms, we use the following parametric probabilistic model for genome. A target genome is modeled as a random vector **s **of length *G *that has the following three key components (a pictorial representation is depicted in Figure 5).

**Random background: **The background of the genome is a random vector, composed of uniformly and independently picked bases from the alphabet set Σ= {*A, C, G, T*}.

**Long flanked repeats: **On top of the random background, we randomly position the longest flanked repeat and the longest flanked triple repeat. Moreover, we randomly position a flanked repeat interleaving the longest flanked repeat, forming the longest pair of flanked interleaved repeat. The corresponding length of the flanked repeats are ${\tilde{\ell }}_{\text{max}}$, ${\tilde{\ell }}_{\text{tri}}$, and ${\tilde{\ell }}_{\text{int}}$ respectively. It is noted that ${\tilde{\ell }}_{\text{max}}>\text{max}\left({\tilde{\ell }}_{\text{int}},{\tilde{\ell }}_{\text{tri}}\right)$.

**Polymorphism and long exact repeats: **Within the repeat interior of the flanked repeats, we randomly position *n*_max_, *n*_int _and *n*_tri _edits (polymorphism) respectively. The sites of polymorphism are chosen such that the longest exact repeat, the longest pair of exact interleaved repeats and the longest exact triple repeat are of lengthℓ_max_,ℓ_int _andℓ_tri _respectively.

## Algorithm design and analysis

### Greedy Algorithm

Read *R*_2 _is a successor of read *R*_1 _if there exists length-*W *suffix of *R*_1 _and length-*W *prefix of *R*_2 _such that they are extracted from the same locus on the genome. Furthermore, there is no other reads that can satisfy the same condition with a larger *W*. To properly determine successors of reads in the presence of long repeats and noise, we need to define an appropriate overlap rule for reads. In this section, we show the conceptual development towards defining such a rule, which is called RA-rule.

**Noiseless reads and long exact repeats: **If the reads are noiseless, all reads can be paired up with their successors correctly with high probability when the read length exceedsℓ_max_. It was done [4] by greedily pairing reads and their candidate successors based on their overlap score in descending order. When a read and a candidate successor are paired, they will be removed from the pool for pairing. Here the overlap score between a read and a candidate successor is the maximum length such that the suffix of the read and prefix of the candidate successor match *exactly*.

**Noisy reads and random background: **Since we cannot expect exact match for noisy reads, we need a different way to define the overlap score. Let us consider the following toy situation. Assume that we have exactly one length-(*ℓ *+ 1) noisy read starting at each locus of a length *G *random genome(i.e. only consists of the random background). Each read then overlaps with its successor precisely by *ℓ *bases. Analogous to the noiseless case, one would expect to pair reads greedily based on overlap score. Here the overlap score between a read and a candidate successor is the maximum length such that the suffix (*x*) of the read and prefix (*y*) of the candidate successor match *approximately*. To determine whether they match *approximately*, one can use a predefined a threshold factor *α *and compute the Hamming distance *d*(*x, y*). If *d*(*x, y*) ≤ *α· ℓ*, then they match *approximately*, otherwise not. Given this decision rule, we can have false positive (i.e. having any pairs of reads mistakenly paired up) and false negative (i.e. having any reads not paired up with the true successors). If false positive and false negative probability are small, this naive method is a reliable enough metric. This can be achieved by using a long enough length *ℓ *>ℓ_iid _and an appropriately chosen threshold *α*.

Recall that ∈is the overall failure probability. By bounding the sum of false positive and false negative probability by *ϵ/*3, one can findℓ_iid _(*p, ϵ/*3, *G*) and *α*(*p, ϵ/*3, *G*) to be the (ℓ_iid_, *α*) solution to the following pair of equations:

$G^{2}\cdot \text{exp}\left(-\ell_{\text{iid}}\cdot D\left(\alpha ∥\frac{3}{4}\right)\right)=\frac{\in }{6}$*(1)*

$G\cdot \text{exp}\left(-\ell_{\text{iid}}\cdot D\left(\alpha ∥2p-\frac{4}{3}p^{2}\right)\right)=\frac{\in }{6}$*(2)*

where $D\left(a∥b\right)=a\text{log}\frac{a}{b}+\left(1-a\right)\text{log}\frac{1-a}{1-b}$ is the Kullback-Leibler divergence.

**Figure 6:**
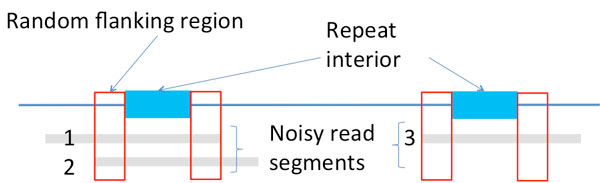
**Intuition about why we define the overlap rule to be RA-overlap rule**.

**Noisy reads and long flanked repeats: **However, when the genome contains long flanked repeats on top of the random background, this naive rule of determining overlap is not enough. Let us look at the example in Figure 6. As shown in Figure 6, because of long flanked repeats, we have a small ratio of overall distance against the overlap length for the segments that are extracted from different copies of the repeat (e.g Segment 1 and Segment 3 in Figure 6). Therefore, the overall Hamming distance between two segments is not a good enough metric for defining overlap. If we abide by the naive rule, we need to increase the read length significantly longer than the flanked repeat length so as to guarantee confidence in deciding approximate match. Otherwise, it will either result in a high false positive rate (if we set a large *α*) or a high false negative rate (if we set a small *α*). To properly handle such scenario, we define a repeat-aware rule(or RA-rule).

*· *RA-matching: Two segments (*x, y*) of length *W *match under the RA-rule if and only if the distance between whole segments is *< α · W *and **both **of its ending segments(of length ℓ_iid_) also have distance <*α · ℓ*_iid_.

*· *RA-overlap: The overlap score between a read and a candidate successor under the RA-rule is the maximum length such that the suffix of the read and prefix of the candidate successor match under the RA-matching.

The RA-rule is particularly useful because it puts an emphasis on both ends of the overlap region. Since the ends are separated by a long range, one end will hopefully originate from the random flanking region of the flanked repeat. If we focus on the segments originating from the random flanking region, the distance per segment length ratio will be very high when the segments originate from different copies of the repeat but very low when they originate from the same copy of the repeat. This is how we utilize the random flank-ing region to differentiate between repeat copies and determine correct successors in the presence of long flanked repeats and noise.

If we use Greedy Algorithm (Alg 1) to merge reads greedily with this overlap rule (RA-rule), Prop 1 shows the information requirement under the previously described sequencing model and genome model. A plot is shown in Figure 1b. Sinceℓ_iid _is of order of tens whereas ${\tilde{\ell }}_{\text{max}}$ is of order of thousands, the read length requirement for Greedy Algorithm to succeed is dominated by ${\tilde{\ell }}_{\text{max}}$. The detailed proof of Prop 1 is given in Appendix.

**Algorithm 1 **Greedy Algorithm

Initialize contigs to be reads

**for ***W *= *L to ℓ*_iid _**do**

  **if**any two contigs x, y are of overlap W under RA-rule

 then

    merge *x, y *into one contig.

  end

end

**Proposition 1 ***With $\ell_{\text{iid}}=\ell_{\text{iid}}\left(p,\frac{\in }{3},G\right)$, if*

$$L>{\tilde{\ell }}_{\text{max}}+2\ell_{iid},$$

$$N>\text{max}\left(\frac{G\;\text{In}\left(3/\in \right)}{L-{\tilde{\ell }}_{\text{max}}-2\ell_{\text{iid}}},\frac{G\;\text{In}\left(3N/\in \right)}{L-{\tilde{\ell }}_{\text{max}}-2\ell_{\text{iid}}}\right)$$

then, Greedy Algorithm(Alg 1) is ϵ - feasible at (N, L).

### Multibridging Algorithm

The read length requirement of Greedy Algorithm has a bottleneck around ${\tilde{\ell }}_{\text{max}}$ because it requires at least one copy of each flanked repeat to be spanned by at least one read for successful reconstruction. Spanning a repeat by a single read is called bridging in [4]. A natural question is whether we need to have all repeats bridged for successful reconstruction.

In the noiseless setting, [4] shows that this condition can be relaxed. Using noiseless reads, one can have successful reconstruction given all copies of each exact triple repeat being bridged, and at least one copy of one of the repeats in each pair of exact interleaved repeats being bridged.

A key idea to allow such a relaxation in [4] is to use a De Bruijn graph to capture the structure of the genome.

When the reads are noisy, we can utilize the random flanking region to specify a De Bruijn graph with high confidence by RA-rule and arrive at a similar relaxation. By some graph operations to handle the residual errors, we can have successful reconstruction with read length ${\tilde{\ell }}_{\text{crit}}+2\cdot \ell_{\text{iid}}<L<{\tilde{\ell }}_{\text{max}}$. The algorithm is summarized in Alg 2. Prop 2 shows its information requirement under the previously described sequencing model and genome model. A plot is shown in Figure 1b. We note that Alg 2 can be seen as a noisy reads generalization of Multibridging Algorithm for noiseless reads in [4].

#### Description and its performance

**Proposition 2 ***With $\ell_{\text{iid}}=\ell_{\text{iid}}\left(p,\frac{\in }{3},G\right)$*, if

$L>{\tilde{\ell }}_{\text{crit}}+2\ell_{\text{iid}},$

$N>\text{max}\left(\frac{G\;In\left(3/\in \right)}{L-{\tilde{\ell }}_{\text{crit}}-2\ell_{\text{iid}}},\frac{G\;In\left(3N/\in \right)}{L-2\ell_{\text{iid}}}\right)$

*then, Multibridging Algorithm(Alg 2) is ϵ - feasible at *(*N, L*).

Detailed proof is given in the Appendix. The following sketch highlights the motivation behind the key steps of Multibridging Algorithm.

[Step1] We set a large K value to make sure the K-mers overlapping the shorter repeat of the longest pair of flanked interleaved repeats and the longest flanked triple repeat can be separated as distinct clusters.

[Step2] Clustering is done using the RA-rule because of the existence of long flanked repeats and noise.

[Step3] A K-mer cluster corresponds to an equivalence class for K-mers matched under the RA-rule. This step forms a De Bruijn graph with K-mer clusters as nodes.

[Step4] Because of large K, the graph can be disconnected due to insufficient coverage. In order to reduce the coverage constraint, we connect the clusters greedily.

[Step5, 7] These two steps simplify the graph. [Step6] Branch clearing repairs any incorrect merges near the boundary of long flanked repeat.

[Step8] Since an Euler path in the condensed graph corresponds to the correct genome sequence, it is traversed to form the reconstructed genome.

#### Some implementation details: improvement on time and space efficiency

For Multibridging Algorithm, the most computational expensive step is the clustering of K-mers. To improve the time and space efficiency, this clustering step can be approximated by performing pairwise comparison of reads.

**Algorithm 2 **Multibridging Algorithm

1. Choose K to be ${\tilde{\ell }}_{\text{crit}}+2\ell_{\text{iid}}$ and extract K-mers from reads.

2. Cluster K-mers based on the RA-rule.

3. Form uncondensed De Bruijn graph *G_De - Bruijn _*= (*V, E*) with the following rule:

a) K-mers clusters as node set *V *.

b) (*u, v*) = *e ϵ E *if and only if there exists K-mers *u*_1 _*ϵ u *and *v*_1 _*ϵ v *such that *u*_1_,*v*_1_are consecutive K-mers in some reads.

4. Join the disconnected components of *G_De-Bruijn _*together by the following rule:

**for ***W *= *K - *1 *to ℓ*_iid _**do**

  **for **each node u which has either no predecessors / successors in G_De-Bruijn _**do**

    a) Find the predecessor/successor *v *for *u *from all possible K-mers clusters such that overlap length(using any representative K-mers in that cluster) between *u *and *v *is *W *under RA-rule.

    b) Add dummy nodes in the De Bruijn graph to link *u *with *v *and update the graph to *G_De-Bruijn _***  end**

end

5. Condense the graph *G_De - Bruijn _*to form *G_string _*with the following rule:

a)Initialize *G_string _*to be *G_De _- _Bruijn _*with node labels of each node being its cluster group index.

b)**while **∃ successive nodes u → v such that out - degree(u) = 1and in - degree(v) = 1 **do**

      bi) Merge *u *and *v *to form a new node *w*

      bii) Update the node label of *w *to be the concatenation of node labels of *u *and *v*

end

6. Clear Branches of *G_string_*:

**for **each node u in the condensed graph G_string _**do**

**if ***out - degree(u)* > 1 *and that all the successive paths are of the same length(measured by the number of node labels) and then joining back to node v and the path length* < ℓ_iid_

    then

we merge the paths into a single path from *u *to *v*.

end

end

7. Condense graph *G_string_*

8. Find the genome :

a)Find an Euler Cycle/Path in *G_string _*and output the concatenation of the node labels to form a string ${\vec{\text{s}}}_{labels}$.

b)Using ${\vec{\text{s}}}_{labels}$ and look up the associated K-mers to form the final recovered genome $\hat{\text{s}}$.

Based on the alignment of the reads, we can cluster K-mers from different reads together using a disjoint set data structure that supports union and find operations. Since only reads are used in the alignment, only the K-mer indices along with their associated read indices and offsets need to be stored in memory-- not all the K-mers.

Pairwise comparison of reads roughly runs in $\tilde{\Theta }\left(N^{2}L^{2}\right)$ if done in the naive way. To speed up the pairwise comparison of noisy reads, one can utilize the fact that the read length is long. We can extract all consecutive *f*-mers (which act as fingerprints) of the reads and do a lexicographical sort to find candidate neighboring reads and associated offsets for comparison. Since the reads are long, if two reads overlap, there should exist some perfectly matched *f*-mers which can be identified after the lexicographical sort. This allows an optimized version of Multibridging Algorithm to run in $\tilde{\Theta }\left(NL\cdot \frac{NL}{G}\right)$ time and $\tilde{\Theta }\left(NLf\right)$ space.

## X-phased Multibridging

As shown in Figure 1b, when long repeats are flanked approximate repeats, there can be a big gap between the noiseless lower bound and the information requirement for Multibridging Algorithm. A natural question is whether this is due to a fundamental lack of information from the reads or whether Multibridging Algorithm does not utilize all the available information. In this section, we demonstrate that there is an important source of information provided by coverage which is not utilized by Multibridging Algorithm. In particular, we introduce X-phased Multibridging, an assembly algorithm that utilizes the information provided by coverage to phase the polymorphism in long flanked repeat interior. The information requirement of X-phased Multibridging is close to the noiseless lower bound (as shown in Figure 1b) even when some long repeats are flanked approximate repeats.

### Description of X-phased Multibridging

Multibridging Algorithm utilizes the random flanking region to differentiate between repeat copies. However, for a flanked approximate repeat, its enclosed exact repeat does not terminate with the random flanking region but only terminates with sparse polymorphism. When we consider the overlap of *two *reads originating from different copies of a flanked approximate repeat, the distinguishing polymorphism is so sparse that it cannot be used to confidently differentiate between repeat copies. Therefore, there is a need to use the extra redundancy introduced by the coverage from *multiple *reads to confidently differentiate between repeat copies and that is what X-phased Multibridging utilizes.

X-phased Multibridging (Alg 3) follows the algorithmic design of Multibridging Algorithm. However, it adds an extra phasing procedure to differentiate between repeat copies of long flanked repeats that Multi-bridging Algorithm cannot confidently differentiate. We recall that after running step 7 of Multibridging Algorithm, a node in the graph *G_string _*corresponds to a substring of the genome and has node label consisting of consecutive K-mer cluster indices. An X-node of *G_string _*is a node that has in-degree and out-degree ≥ 2. X-node indeed corresponds to a flanked repeat. The incoming/outgoing nodes of the X-node correspond to the incoming/outgoing random flanking region of the flanked repeat.

To be concrete, we focus the discussion on a pair of flanked interleaved repeats, assuming triple repeats are not the bottleneck. However, the ideas presented can be generalized to repeats of more copies.

**Figure 7:**
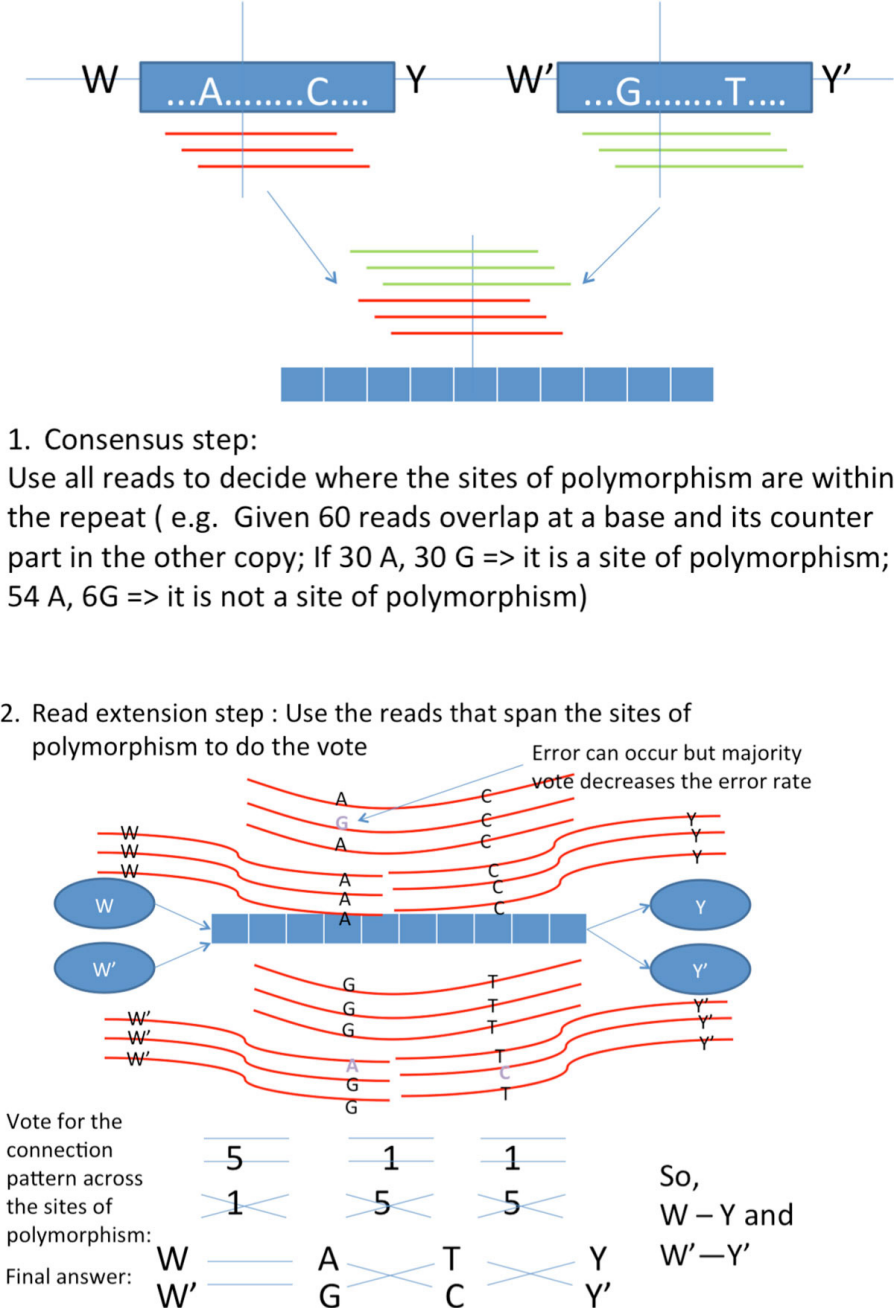
**Illustration of how to phase polymorphism to extend reads across repeats**.

For the flanked approximate repeat with length ℓ_int _*< L *and ${\tilde{\ell }}_{\text{int}\;}>L$ (as shown in Figure 7), there is no node-disjoint paths joining incoming/outgoing random flanking region with the distinct repeat copies in *G_string_*. It is because the reads are corrupted by noise and the polymorphism is too sparse to differentiate between the repeat copies. Executing Multibridging Algorithm directly will result in the formation of an X-node, which is an artifact due to K-mers from different copies of the flanked approximate repeat erroneously clustered together.

Successful reconstruction requires an algorithm to pair up the correct incoming/outgoing nodes of the X-node(i.e. decide how $W,W^{\prime }$ and $Y,Y^{\prime }$ are linked in Figure 7). This is handled by the phasing procedure in X-phased Multibridging, which uses all the reads information. The phasing procedure is composed of two main steps:

Consensus step: Confidently find out where the sites of polymorphism are located within the flanked repeat interior.

Read extension step: Confidently determine how to extend reads using the random flanking region and sites of polymorphism as anchors.

**Consensus step **For the X-node of interest, let *D *be the set of reads originating from any sites of the associated flanked repeat region and let *x*_1 _and *x*_2 _denote the associated repeat copies. Since the random flanking region is used as anchor, it is treated as the starting base (i.e. *x*_1_(0) = *W *and $x_{2}\left(0\right)=W^{\prime }$). For the *i^th ^*subsequent site of the flanked repeat (where $1\leq i\leq {\tilde{\ell }}_{\text{int}\;}$), we determine the consensus according to Eq (3). This can be implemented by counting the frequency of occurrence of each alphabet overlapping at each site of the repeat. The consensus result determines the sites of polymorphism and the most likely pairs of bases at the sites of polymorphism.

$\underset{F\subset {\left\{A,C,G,T\right\}}^{2}}{\text{max}}P\left(\left\{x_{1}\left(i\right),x_{2}\left(i\right)\right\}=F|D\right)$*(3)*

**Read extension step **After knowing the sites of polymorphism, we use those reads that span the sites of polymorphism or random flanking region to help decide how to extend reads across the flanked repeat. Let *σ *be the possible configuration of alphabets at the sites of polymorphism and random flanking region (e.g. $\sigma =\left(ACY,GTY^{\prime }\right)$ means that the two copies of the flanked repeat with the corresponding random flanking region respectively are W-A-C-Y, W'-G-T-Y' where the common bases are omitted).

**Algorithm 3 **X-phased Multibridging

1. Perform Step 1 to Step 7 of MultiBridging Algorithm

2. For every X-node *x ϵ G_string_*

a)Align all the relevant reads to the flanked repeat *x*

b)Consensus step: Consensus to find location of polymorphism by solving Eq (3)

c)Read extension step: If possible, resolve flanked repeat(i.e. pair up the incoming/outgoing nodes of *x*) by either countAlg or by solving Eq (4)

3. Perform Step 8 of MultiBridging Algorithm as in Alg 2

The following maximum a posteriori estimation is used to decide the correct configuration.

$$\underset{\sigma }{\text{max}}P\left(\hat{\sigma }=\sigma |D,{\left\{x_{1}\left(i\right),x_{2}\left(i\right)\right\}}_{i=1}^{{\tilde{\ell }}_{\text{int}\;}}\right)$$

where $\hat{\sigma }$ is the estimator, *D *is the raw read set, and *x*_1_, *x*_2 _are the estimates from the consensus step. It is noted that the size of the feasible set for *σ *is ${{\text{2}}^{n}}^{{}_{\text{int}}+\text{1}}$.

In practice, for computational effciency, the maximization in Eq (4) can be approximated accurately even if it is replaced by the simple counting illustrated in Figure 7, which we call count-to-extend algorithm(countAlg). CountAlg uses the raw reads to establish majority vote on how one should extend to the next sites of polymorphism using only the reads that span the sites of polymorphism.

### Performance

After introducing the phasing procedure in X-phased Multibridging, we proceed to find its information requirement for successful reconstruction.

The information requirement for X-phased Multibridging is the amount of information required to reduce the error of the phasing procedure to a negligible level. The phasing procedure - step 2 in Alg. 3 - is a combination of consensus and read extension steps, which contribute to the error as follows.

Let *ε *be the error event of the repeat phasing procedure for a repeat, *ϵ*_1 _be the error probability for the consensus step, *ϵ*_2 _be the error probability for the read= extension step given *k *reads spanning each consecutive site of polymorphism within the flanked repeat, *δ_cov _*be the probability for having *k *reads spanning each consecutive sites of polymorphism(i.e. *k *bridging reads) within the flanked repeat. We have,

$P\left(\epsilon \right)\leq \in_{1}+\in_{2}+\delta_{\text{cov}\;}$*(5)*

Therefore, to guarantee confidence in the phasing procedure, it suffices to upper bound *ϵ*_1_,*ϵ*_2 _and *δ_cov_*. We tabulate the error probabilities of *ϵ*_1_, *ϵ*_2 _in Table 1 for phasing a flanked repeat (whose length is 5000 whereas the genome length is 5M). The flanked repeat has two sites of polymorphism which partition it into three equally spaced segments.

**Table 1:** Calibration of error probability made by the phasing procedure of X-phased Multibridging

*p*	Coverage (NL/G)	*ϵ*_1_
0.01	20	0.00

0.01	40	0.00

0.01	60	0.00

0.1	20	0.16

0.1	40	0.00

0.1	60	0.00

(a) Calibration for *ϵ*_1._		

***p***	**Number of bridging reads *k***	**Upper bound for *ϵ*_2_**

0.01	1	0.060

0.01	3	0.0036

0.01	5	0.00024

0.1	11	0.089

0.1	21	0.022

0.1	31	0.0059

(b) Calibration for *ϵ*_2_		

From Table 1, when *p *= 0.01, the information requirement translates to the condition of having three bridging reads spanning the shorter exact repeat of the longest pair of exact interleaved repeats. Therefore, the information requirement for X-phased Multibridging shown in Figure 1b also corresponds to this condition. It is noted that X-phased Multibridging has the same vertical asymptote as the noiseless lower bound. The vertical shift is due to the increase of requirement on the number of bridging reads from *k *= 1 (noiseless case) to *k *= 3 (noisy case).

## Simulation of the prototype assembler

**Figure 8:**
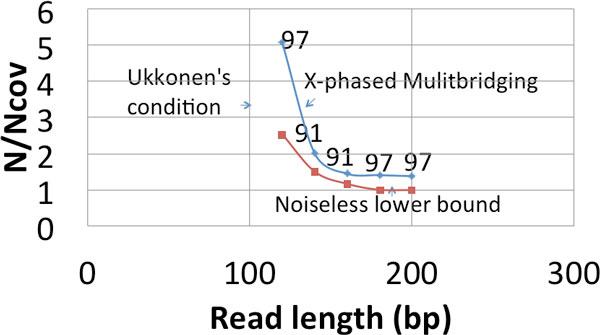
**Simulation results on the assembly of synthetic genomes using reads corrupted by substitution noise. The parameters are as follows. $G=10k;{\tilde{\ell }}_{\text{max}}=\ell_{\text{max}}=500$, ${\tilde{\ell }}_{\text{int}\;}=200$, ℓ_int _= 100 with two sites of polymorphism within the flanked repeat. *p*= 1.5%, ∈= 5%**.

Based on the algorithmic design presented, we implement a prototype assembler for automatic genome finishing using reads corrupted by substitution noise. First, the assembler was tested on synthetic genomes, which were generated according to the genome model described previously. This demonstrates a proof-of-concept that one can achieve genome finishing with read length close toℓ_crit_, as shown in Figure 8. The number on the line represents the number of simulation rounds (out of 100) in which the reconstructed genome is a single contig with ≥ 99% of its content matching the ground truth.

Second, the assembler was tested using synthetic reads, sampled from genome ground truth downloaded from NCBI. The assembly results are shown in Table 2. The observation from the simulation result is that we can assemble genomes to finishing quality with information requirement near the noiseless lower bound. More information about the detail design of the prototype assembler is presented in the Appendix and source code/data set can be found in [19].

**Table 2:** Simulation results on the assembly of several real genomes using reads corrupted by substitution noise ((a) *Prochlorococcus marinus *(b) *Helicobacter pylori *(c) *Methanococcus maripaludis *(d) *Mycoplasma agalactiae*)withℓ_crit _= max(ℓ_int_,ℓ_tri_), ${\tilde{\ell }}_{\text{crit}}=\text{max}\left({\tilde{\ell }}_{\text{int}},{\tilde{\ell }}_{\text{tri}}\right)$ and *N_noiseless _*is the lower bound on number of reads in the noiseless case for 1 *- ϵ *= 95% confidence recovery

**Index**	**Species**	***G***	***p***	$\frac{NL}{G}$	***L***	${\tilde{l}}_{\text{max}}$	${\tilde{l}}_{\text{crit}}$	**ℓ_crit_**	**% match**	**Ncontig**	$\frac{N}{N_{noiseless}}$	$\frac{L}{\ell_{\text{crit}}}$
1	a	1440371	1.5%	37.36 X	930	1817	803	770	100.00	1	1.57	1.21
2	a	1440371	1.5%	33.14 X	970	1817	803	770	99.95	1	1.67	1.26
3	a	1440371	1.5%	29.60 X	1000	1817	803	770	99.99	1	1.66	1.30
4	b	1589953	1.5%	40.82 X	2440	4183	2155	2122	100.00	1	1.30	1.15
5	b	1589953	1.5%	21.31 X	2752	4183	2155	2122	99.99	1	1.19	1.30
6	b	1589953	1.5%	20.66 X	2900	4183	2155	2122	99.99	1	1.35	1.37
7	c	1772693	1.5%	30.03 X	3950	5018	3234	3218	99.96	1	1.36	1.23
8	c	1772693	1.5%	21.96 X	4279	5018	3234	3218	99.97	1	1.33	1.33
9	c	1772693	1.5%	17.03 X	4700	5018	3234	3218	100.00	1	1.31	1.46
10	d	1006701	1.5%	35.23 X	6867	15836	10518	5494	99.05	1	1.72	1.25
11	d	1006701	1.5%	19.88 X	7500	15836	10518	5494	97.86	1	1.30	1.37
12	d	1006701	1.5%	17.69 X	9000	15836	10518	5494	98.10	1	1.68	1.64

## Extension to handle indel noise

A further extension of the prototype assembler addresses the case of reads corrupted by indel noise. Similar to the case of substitution noise, tests were performed on synthetic reads sampled from real genomes and synthetic genomes. Simulation results are summarized in Table 3 where *p_i_, p_d _*are insertion probability and deletion probability and rate is the number of successful reconstruction(i.e. simulation rounds that show mismatch < 5%) divided by total number of simulation rounds. The simulation result for indel noise corrupted reads shows that X-phased Multibridging can be generalized to assemble indel noise corrupted reads. The information requirement for automated finishing is about a factor of two from the noiseless lower bound for both N and L.

**Table 3:** Simulation results on the assembly of real/synthetic genomes using reads corrupted by indel noise(Synthetic: randomly generated to fit ${\tilde{\ell }}_{\text{max}}$, ${\tilde{\ell }}_{\text{crit}}$, ℓ_crit_; (a) : *Prochlorococcus marinus *; (b): *Helicobacter pylori*)

**Type**	***G***	***p_i_***	***p_d_***	${\tilde{\ell }}_{\text{crit}}$	***L***	$\frac{NL}{G}$	${\tilde{\ell }}_{\text{max}}$	**ℓ_crit_**	${\tilde{\ell }}_{\text{crit}}$	$\frac{N}{N_{noiseless}}$	**Rate**
Synthetic	50000	1.5%	1.5%	23.0 X	200	500	200	100	2.25	2	28/30
Synthetic	50000	1.5%	1.5%	24.1 X	180	500	200	100	2.33	1.8	27/30
a	1440371	1.5%	1.5%	28.53 X	1000	1817	803	770	1.60	1.30	1/1
b	1589953	1.5%	1.5%	20.66 X	2900	4183	2155	2122	1.35	1.37	1/1

a

**Figure 9:**
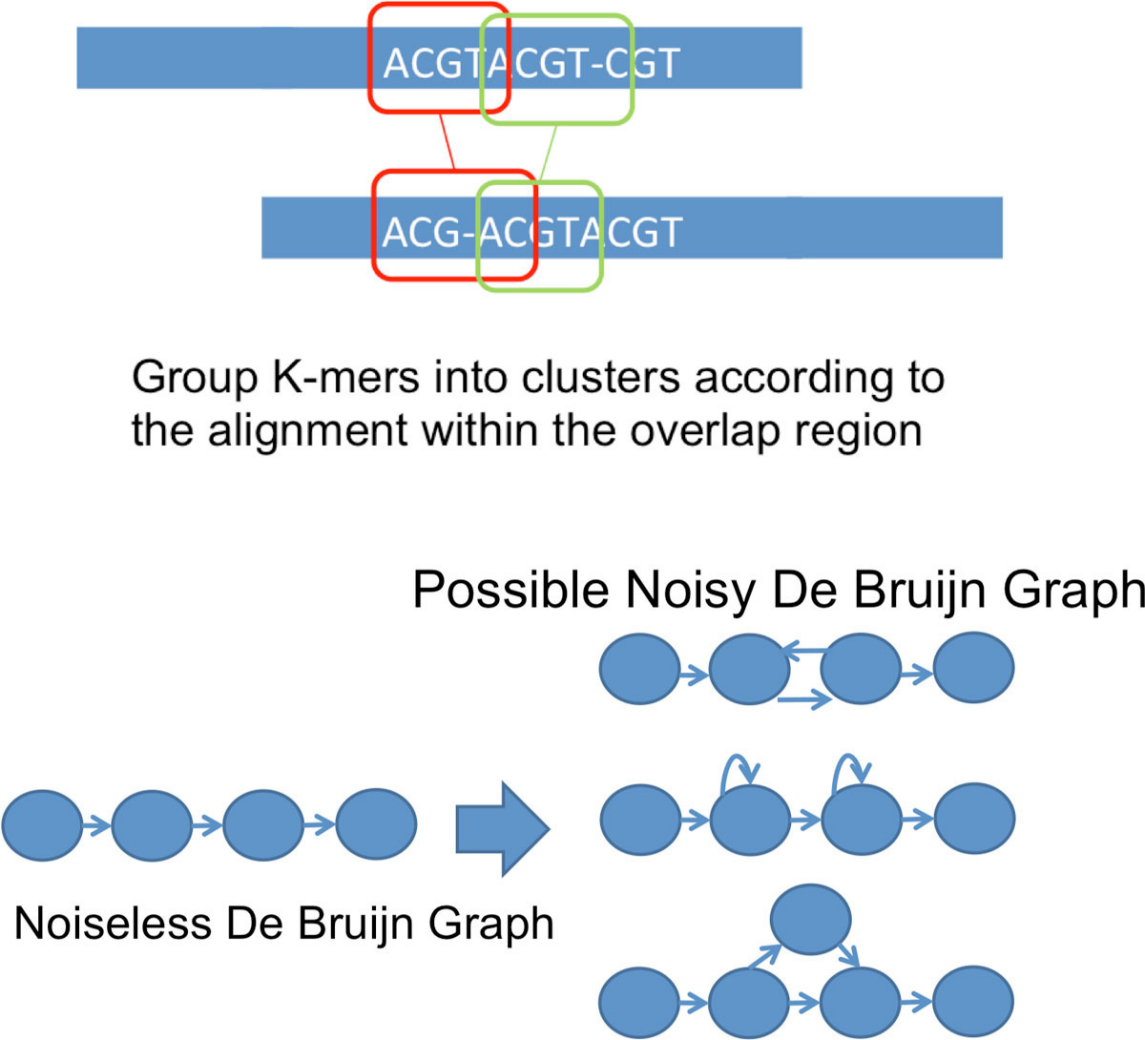
Treatment of reads corrupted by indel noise

We remark that one non-trivial generalization is the way that we form the noisy De Bruijn graph for K-mer clusters. In particular, we first compute the pairwise overlap alignment among reads, then we use the overlap alignment to group K-mers into clusters. Subsequently, we link successive cluster of K-mers together as we do in Alg 2. An illustration is shown in Figure 9a. However, due to the noise being indel in nature, the edges in the noisy De Bruijn graph may point in the wrong direction as shown in Figure 9b. In order to handle this, we traverse the graph and remove such abnormality when they are detected.

## Conclusion

In this work, we show that even when there is noise in the reads, one can successfully reconstruct with information requirements close to the noiseless fundamental limit. A new assembly algorithm, X-phased Multi-bridging, is designed based on a probabilistic model of the genome. It is shown through analysis to perform well on the model, and through simulations to perform well on real genomes.

The main conclusion of this work is that, with an appropriately designed assembly algorithm, the information requirement for genome assembly is insensitive to moderate read noise. We believe that the information theoretic insight is useful to guide the design of future assemblers. We hope that these insights allow future assemblers to better leverage the high throughput sequencing read data to provide higher quality assembly.

## Competing interests

The authors K.K.L and A.K are or were employees of Pacific Biosciences, a company commercializing DNA sequencing technologies at the time that this work was completed.

## Authors' contributions

K.K.L, A.K and D.T performed research and wrote the manuscript. K.K.L implemented the algorithms and performed the experiments.

## Additional file 1

Details of the proofs, in-depth description of the design of the prototype assembler and details of simulation results are presented.

## Declarations

The authors K.K.L and D.T. are partially supported by the Center for Science of Information (CSoI), an NSF Science and Technology Center, under grant agreement CCF-0939370.

This article has been published as part of BMC Bioinformatics Volume 15 Supplement 9, 2014: Proceedings of the Fourth Annual RECOMB Satellite Workshop on Massively Parallel Sequencing (RECOMB-Seq 2014). The full contents of the supplement are available online at http://www.biomedcentral.com/bmcbioinformatics/supplements/15/S9.
